# Mortality Benefit of Tranexamic Acid for Hemorrhage With Concurrent Traumatic Brain Injury: Outcomes From a Prospective Cohort Study in a High‐Trauma, Prolonged Care Setting

**DOI:** 10.1002/wjs.70161

**Published:** 2025-11-28

**Authors:** Julia M. Dixon, Adane F. Wogu, Maria D. Rodriguez, Dale Barnhart, Rachel Patel, Hendrick J. Lategan, George Oosthuizen, Janette Verster, Shaheem de Vries, Craig Wylie, EpiC Study Site Collaborators, Elaine Erasmus, Steven G. Schauer, Nee‐Kofi Mould‐Millman

**Affiliations:** ^1^ Department of Emergency Medicine School of Medicine University of Colorado, Anschutz Medical Campus Aurora Colorado USA; ^2^ Department of Biostatistics and Informatics Colorado School of Public Health University of Colorado Anschutz Medical Campus Aurora Colorado USA; ^3^ Division of Surgery Department of Surgical Sciences Stellenbosch University Cape Town South Africa; ^4^ Division of Forensic Medicine, Department of Pathology Stellenbosch University Cape Town South Africa; ^5^ Collaborative for Emergency Care in Africa Cape Town South Africa; ^6^ Emergency Medical Services Western Cape Government Health Bellville South Africa; ^7^ Western Cape Government Health and Wellness Cape Town South Africa; ^8^ Division of Emergency Medicine Department of Family and Emergency Medicine Stellenbosch University Cape Town South Africa; ^9^ US Army Institute of Surgical Research JBSA Fort Sam Houston Texas USA

**Keywords:** emergency medicine, glasgow outcomes score, hemorrhage, multiple organ failure, tranexamic acid, trauma, traumatic brain injury

## Abstract

**Background:**

Traumatic brain injury (TBI) and hemorrhage are leading causes of trauma death and disability worldwide. The concurrence of hemorrhage and brain injury carries a two‐fold increase in mortality and clinical management of patients with concurrent TBI and hemorrhage is challenging. Tranexamic acid (TXA) has been shown to reduce mortality from hemorrhage and TBI independently, however there is sparse evidence on the potential benefit of TXA in patients with both non‐head hemorrhage and TBI.

**Methods:**

We conducted a secondary database analysis of EpiC, a multicenter, prospective cohort of trauma patients in South Africa. We compared the morbidity and mortality of patients experiencing both non‐head hemorrhage and TBI who received TXA within 3‐h post‐injury versus similarly injured patients who did not receive TXA. Inverse probability treatment weighting (IPTW) was implemented followed by a multivariable logistic regression to evaluate 7‐day mortality. Secondary outcomes included the worst 7‐day sequential organ failure assessment (SOFA) and neurologic recovery assessed by Glasgow Outcomes Score Extended (GOSE).

**Results:**

A total of 656 patients were included in the analysis. 132 (20%) received TXA within 3 h and 544 (80%) did not. For the primary outcome of 7‐day mortality, treatment with TXA was associated with a 22% reduction in odds of death (mOR, 0.78, 95% CI, 0.62–0.98). TXA‐treated patients had significant lower odds of SOFA > 4 or death (mOR, 0.71; 95%CI, 0.53–0.95) and non‐significantly reduced odds of poor functional status at 3 months (GOSE < 7 or death) (mOR, 0.89; 95% CI, 0.68–1.18). Treatment with TXA within 2 h was associated with a 27% reduction in odds of 7‐day mortality (mOR, 0.73; 95%CI, 0.61–0.86).

**Conclusions:**

In this study, the administration of TXA within 3 h to patients with concurrent hemorrhage and TBI was associated with a 22% reduction in mortality at 7 days. The mortality benefit was slightly larger when TXA was given within 2 h. TXA treatment was also associated with lower risk of organ failure. These results support a growing body of evidence that TXA is an effective intervention to reduce mortality and morbidity after traumatic injury.

## Graphical abstract

Early TXA administration (≤ 3 h) in concurrent hemorrhage and TBI was significantly associated with a 22% reduction in mortality at 7 days. TXA treatment was also associated with a significant 29% lower odds of organ failure and non‐significant 11% odds of good neurologic recovery.

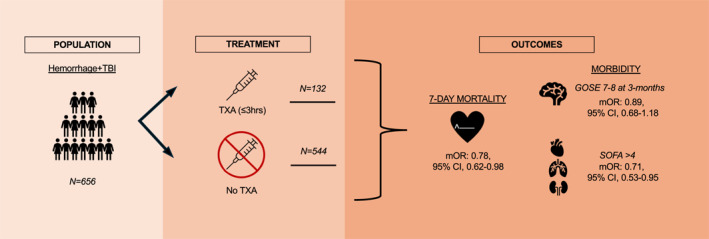

## 1 Introduction

Injury remains a leading global cause of mortality, accounting for over 4.4 million annual deaths with traumatic brain injury (TBI) and uncontrolled hemorrhage representing the most fatal causes across civilian and military populations [1, 2, 3]. Approximately 3.2 million deaths are attributed to unintentional injuries while an estimated 1.3 million deaths result from violence‐related injuries [1]. Trauma is a major public health issue in low‐ and middle‐income countries, and particularly in South Africa where injury ranks as the third leading cause of death among adults aged 33–44 and contributes significantly to long‐term disability [1, 4].

Patients with multiple system or multi‐organ injuries, including those with both hemorrhage and TBI, account for approximately 25% of major trauma admissions in high income countries, and 30% of all major trauma cases in middle‐income countries [5, 6]. Globally, over 25% of TBI patients also present with uncontrolled extra‐cranial hemorrhage, with reported mortality rates approximately 10‐fold higher than those with neither condition and significantly higher than those with either TBI or hemorrhagic shock [7, 8]. Patients with concurrent hemorrhage outside the head and TBI (hemorrhage + TBI) are at highest risk for poor outcomes given dual insults with competing physiology and challenging clinical management [8, 9]. Hemorrhage causes low blood pressure, shock, and trauma‐induced coagulopathy which in combination can worsen TBI progression due to reduced intra‐cranial oxygenation and perfusion [8, 10, 11]. Common physiology in both traumatic hemorrhage and TBI are the presence of coagulopathy and pro‐inflammatory states [12, 13, 14]. Consequently, patients with hemorrhage + TBI experience the highest mortality rates with the risk for poor outcomes being highest in resource‐strained settings [15].

Further scientific evidence for therapeutics that impact both trauma‐induced coagulopathy and immune dysregulation is needed. Tranexamic (TXA) is an antifibrinolytic medication that has demonstrated effectiveness in traumatic hemorrhage management by competitively inhibiting the activation of plasminogen to plasmin, thereby reducing fibrin degradation and stabilizing formed clots [16, 17]. Additionally, TXA's ability to decrease fibrinolysis helps minimize the inflammatory response associated with hemorrhagic shock, possibly reducing the risk of multiple organ dysfunction [18, 19]. Findings presented by the Clinical Randomization of Antifibrinolytic in Significant Hemorrhage (CRASH)‐2 and CRASH‐3 trials showed that administration of TXA within 3 h of injury reduced 28‐day mortality among patients at risk for hemorrhage and among patients with mild‐to‐moderate traumatic brain injuries, respectively [20, 21]. Further, most TXA studies indicate a time‐dependence with early administration showing improved survival in patients with traumatic hemorrhage, with or without TBI [16, 17, 22, 23]. TXA is particularly advantageous in resource‐limited settings because it is relatively inexpensive, shelf‐stable, and has a strong safety profile [24, 25].

Most TXA studies have examined either torso or intracranial hemorrhage, with few addressing patients with both [22, 26, 27, 28, 29, 30]. Further, the majority of TXA outcomes evidence is largely drawn from studies conducted in urban, well‐resourced settings with rapid access to definitive care, resulting in limited generalizability to low‐resourced and austere settings, or those with significant delays to care [31, 32, 33]. Additionally, morbidity outcomes such as organ failure and neurotrauma outcomes remain underexplored. We assess differences in mortality and morbidity among injured patients with hemorrhage + TBI who received TXA within 3 h of injury compared to patients with comparable injuries that did not receive TXA in a resource‐constrained, high‐trauma system in South Africa.

## 2 Methods

### 2.1 Design

We conducted a secondary analysis using data collected from the Epidemiology and Outcomes of Combat‐Relevant Prolonged Trauma Care (EpiC) study. The EpiC study is a prospective, multicenter cohort study of adult trauma patients in Western Cape, South Africa. Data from the EpiC study links pre‐ and in‐hospital care with mortuary reports through standardized clinical chart abstraction from the patient's trajectory through the health system after an injury. EpiC patients are enrolled from 12 sites in the Western Cape Province including four emergency medical service bases, six public hospitals (ranging from primary to tertiary care in both urban and rural areas), and two forensic pathology laboratories [34].

This study received ethical approval with a waiver of informed consent from the Human Research Ethics Committee of Stellenbosch University (Ethics Reference No. N20/03/036), a reliance agreement from the Colorado Multiple Institute Review Board (COMIRB #20–2176) and a concurrence memo from the Defense Health Agency Office of Human Research Oversight with the U.S. Department of Defense's subject protection requirements (OHRO Log #E01863.1x).

### 2.2 Population/Setting

The Western Cape Province of South Africa is a high‐trauma, resource‐constrained setting shaped by gang violence, substance abuse, and socio‐economic disparities [35, 36]. Injured patients receive initial resuscitation and stabilization at the health care facility closest to the point of injury which is often a district hospital or community health center, capable of delivering critical airway, breathing and circulatory interventions, consistent with international guidelines, but with limited blood products, diagnostics and access to surgical care [34]. Patients are then transferred to regional and/or tertiary facilities for both advanced diagnostics and specialized trauma care.

### 2.3 Inclusion/Exclusion

EpiC includes adult trauma patients (≥ 18 years of age) who sustained injuries within 24 h and who were alive (i.e., had signs of life or attempted resuscitation) prior to their first contact with the healthcare system (emergency medical services (EMS) or hospital). Exclusions include prisoners or individuals with trauma secondary to drowning, envenomation, electrocution, or bites/stings.

**FIGURE 1:**
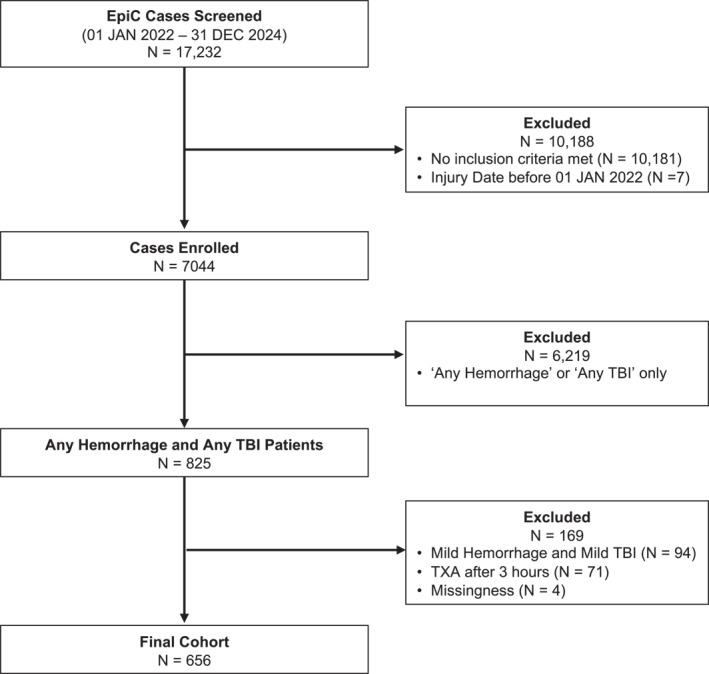
Inclusion‐exclusion flow diagram.

This sub‐study includes patients who presented to an EpiC study site between 01 January 2022 to 31 December 2024 with blunt and/or penetrating mechanisms of injury and with evidence of concurrent hemorrhage + TBI. We identified TBI with a head body region abbreviated injury score (AIS) severity of ≥ 2 or death from a head injury. We defined hemorrhage with at least one non‐head, non‐face AIS injury and at least one shock vital sign or laboratory result, hemorrhage diagnosis or intervention (e.g., blood products) or death from hemorrhage. (Supporting Information S1) Severity (mild, moderate or severe) of TBI was classified using Glasgow Coma Scale (GCS) and computed tomography (CT) findings and severity for hemorrhage by vital signs and blood product volumes. (Supporting Information S2) Patients meeting both mild TBI and mild hemorrhage or who were administered TXA > 3 h after injury or were missing data on other key variables were excluded (Figure 1).

### 2.4 Exposure, Outcomes, and Key Variables

Our study compared patients who received intravenous TXA (any dose) within 3 h of injury compared to those who did not receive TXA at any time during hospital admission. Our primary outcome was 7‐day mortality. Secondary outcomes included 48‐h, 72‐h, and 30‐day hospital mortality, multiple organ failure (MOF) as measured by worst sequential organ failure assessment (SOFA) scores during the first 7 days of admission and dichotomized as either no or mild MOF (none or SOFA ≤ 4) or MOF (SOFA > 4 or death), and functional outcomes assessed via Glasgow Outcome Scale‐Extended (GOSE) at 3‐month post‐discharge dichotomized as either good recovery (GOSE ≥ 7) or poor recovery (GOSE < 7 or death) [37]. We used a hierarchical substitution strategy to address missing data for GOSE 3‐month post‐discharge using available data from the 6‐month, 6‐week, 1‐year, Glasgow Outcome at Discharge Score (GODS) assessment and discharge GCS in that order. (Supporting Information S1).

Other variables of interest included patient demographics (sex, age); injury characteristics (force type, mechanism), and measures of pre‐treatment injury severity (initial shock index, initial systolic blood pressure), AIS scores, New Injury Severity Score (NISS), initial GCS.

### 2.5 Analysis

We described our study population with counts and frequencies for categorical variables and medians and interquartile ranges for continuous variables. We used inverse probability treatment weighting (IPTW) to reduce confounding by balancing patient characteristics between TXA‐treated and untreated groups. Pre‐intervention covariates in our propensity score (PS) model were guided by clinical and subject‐matter expertise prioritizing inclusion of known confounders (variables associated with both treatment and outcome) and outcome predictors while excluding post‐treatment variables to avoid collider bias (Supporting Information S1) [38, 39]. Standardized mean difference (SMD) was used to evaluate whether adequate balance was achieved between the two groups.

We used separate weighted multivariable logistic regression models to evaluate the association between TXA administration and each primary and secondary outcome. IPTW and weighted multivariable logistic regression model covariates are listed in Supporting Information S1. To account for correlation among patients treated within the same hospital, we estimated cluster‐robust standard errors by hospital [40]. Results are presented as marginal odds ratios (mOR) with 95% confidence intervals (CI).

For 30‐day mortality, we additionally applied a Cox proportional hazards (PH) model to incorporate event timing and censoring. Survival was defined as time from admission to death within 30 days, with TXA administration modeled as a time‐dependent covariate to mitigate immortal time bias [41]. A weighted and covariate‐adjusted model was fitted using the same variables as in the multivariable logistic regression, with results reported as hazard ratio (HR) with 95% CI. Proportional hazards assumption was formally evaluated using Schoenfeld residuals [42, 43].

Pre‐planned subgroup analysis included: (1) TXA treatment within 2 h of injury, (2) TXA treatment within 1 h of injury, (3) removal of patients that died due to catastrophic tissue destruction (CTD) (e.g., non‐survivable injuries), (4) removal of severe TBI patients. Additional sensitivity analyses included using an interaction term to assess associations by injury force type (blunt vs. penetrating vs. blunt and penetrating) and severity (mild/moderate vs. severe) of hemorrhage, TBI and hemorrhage + TBI. For each subgroup and sensitivity analysis, we refit the IPTW models and estimated odds ratios using multivariable logistic regression in the subgroups.

All analyses were conducted using R (version 4.5.0), utilizing the PS weight and marginal effects‐related packages for tasks such as weighting, covariate balance assessment, and average marginal effect estimation. Statistical tests were performed at a 5% significance level [44, 45].

## 3 Results

A total of 656 patients met inclusion criteria and all were retained after IPTW. Most patients were male (567, 86%) with a median age of 32.2 (26.5, 38.0). The most frequent injury force type was blunt (451, 69%). A total of 132 (20%) patients received TXA within 3 h of injury and 544 (80%) received no TXA.

Patients who received TXA had more severe injuries than those who did not with a higher median NISS (34.0 vs. 27.0), more severe brain injury with a GCS ≤ 8 (63, 48% vs. 154, 29%) and more serious‐to‐severe neck or torso injuries as categorized by AIS (62, 47% vs. 139, 27%) (Table 1). After IPTW patients were adequately balanced, as indicated by an SMD value < 0.10.

**TABLE 1:** Unweighted patient characteristics by treatment group with SMD values after IPTW.

Characteristics	No TXA (*N* = 524)	TXA (3h) (*N* = 132)	SMD^a^
Sex, male^b^, *n* (%)	455 (86.8)	112 (84.8)	0.02
Age (in years)^b^, median [IQR]	32.0 [26.0, 37.6]	33.2 [27.3, 39.0]	0.01
Injury force type, *n* (%)			
Penetrating	88 (16.8)	33 (25.0)	0.05
Blunt	367 (70.0)	84 (63.6)	0.13
Blunt + penetrating	69 (13.2)	15 (11.4)	0.08
Injury mechanism^b^, *n* (%)			
Stabbing/cut	83 (15.8)	18 (13.6)	0.06
Struck/hit	224 (42.7)	20 (15.2)	0.05
Firearm/gunshot	38 (7.3)	21 (15.9)	0.00
Vehicular	167 (31.9)	72 (54.5)	0.00
Other	12 (2.3)	1 (0.8)	0.01
Shock index^b^, median [IQR]	0.9 [0.7, 1.1]	1.0 [0.7, 1.2]	0.01
Systolic blood pressure, *n* (%)			
Hypotension (SBP ≤ 90)	121 (23.2)	36 (27.3)	0.01
Normal (SBP > 90–120)	314 (60.2)	78 (59.1)	0.01
Hypertension (SBP ≥ 120)	87 (16.7)	18 (13.6)	0.00
New injury severity score^b^, median [IQR]	27.0 [17.0, 38.0]	34.0 [26.8, 43.0]	0.05
Glasgow coma scale^b^, *n* (%)			
Severe (≤ 8)	154 (29.4)	63 (47.7)	0.00
Moderate (9–12)	86 (16.4)	22 (16.7)	0.00
Mild (13–15)	275 (52.5)	44 (33.3)	0.01
Missing	9 (1.7)	3 (2.3)	0.00
Received blood before treatment^b^, *n* (%)	50 (9.5)	37 (28.0)	0.03
Received blood after treatment window^c^, *n* (%)	0 (0.0)	29 (22.0)	0.17
AIS: Head^b^, *n* (%)			
1–2, minor‐moderate	86 (16.4)	16 (12.1)	0.00
3–4, serious‐severe	320 (61.1)	83 (62.9)	0.07
5–6, critical‐maximal	118 (22.5)	33 (25.0)	0.06
AIS: Neck and torso^b^, *n* (%)			
None/unknown	227 (43.3)	33 (25.0)	0.02
1–2, minor‐moderate	138 (26.3)	27 (20.5)	0.04
3–4, serious‐severe	139 (26.5)	62 (47.0)	0.01
5–6, critical‐maximal	20 (3.8)	10 (7.6)	0.00
AIS: All other regions^b^, *n* (%)			
None/unknown	38 (7.3)	17 (12.9)	0.00
1–2, minor‐moderate	366 (69.8)	64 (48.5)	0.03
3–4, serious‐severe	111 (21.2)	44 (33.3)	0.03
5–6, critical‐maximal	9 (1.7)	7 (5.3)	0.00
TXA dose^d^			
1 g	n/a	86 (65.2)	
2 g	n/a	43 (32.6)	
Time to TXA (hr), median [IQR]		1.5 [0.9, 2.2]	
Time from injury to 1st facility < 3 h, yes^b^, *n* (%)	257 (49.0)	69 (52.3)	0.06
Discharge GCS, median [IQR]	15.0 [15.0, 15.0]	15.0 [15.0, 15.0]	0.06

Abbreviations: AIS: Abbreviated injury score, GCS: Glasgow coma scale, TXA: tranexamic acid.

^a^ SMD: Standardized mean difference.

^b^ Covariates were included in the propensity score modeling.

^c^ Treatment window in untreated group defined as blood > 3 h from injury, in TXA treated group defined as blood after TXA treatment, no patients in the “No TXA” group received blood > 3 h after injury.

^d^ One patient received each of the following doses 0.5, 3 or 4 g within the 3‐h treatment window.

Unadjusted overall mortality was 33%, the TXA group experienced higher 7‐day mortality (33.3% vs. 27.5%), higher rates of MOF (62.1%, vs. 46.2%) and a higher rate of poor recovery (56.8% vs. 40.3%) (Table 2).

**TABLE 2:** Unadjusted primary and secondary outcomes overall and by treatment group.

Outcomes	Overall (*N* = 656)	No TXA (*N* = 524)	TXA (3h) (*N* = 132)
Mortality			
48‐h	139 (21.2%)	108 (20.6%)	31 (23.5%)
72‐h	153 (23.3%)	120 (22.9%)	33 (25.0%)
7‐day^a^	188 (28.7%)	144 (27.5%)	44 (33.3%)
30‐day	214 (32.6%)	164 (31.3%)	50 (37.9%)
Worst SOFA score first 7 days			
No MOF	332 (50.6%)	282 (53.8%)	50 (37.9%)
MOF	324 (49.4%)	242 (46.2%)	82 (62.1%)
GOSE (3m post‐discharge)			
Good recovery	370 (56.4%)	313 (59.7%)	57 (43.2%)
Poor recovery	286 (43.6%)	211 (40.3%)	75 (56.8%)

Abbreviations: GOSE, Glasgow outcome scale: extended; MOF, multi‐organ failure.

^a^ Primary outcome.

For the primary outcome of 7‐day mortality, TXA treatment was associated with a significant 22% reduction in odds of death (mOR 0.78, 95% CI 0.62–0.98) (Table 3). Secondary mortality outcomes demonstrated similar beneficial associations with a 26% reduction in odds of 48‐h mortality (mOR 0.74; 95%CI 0.61–0.90) and a 33% reduction in 72‐h mortality (mOR 0.67; 95%CI 0.61–0.74) but a non‐significant 14% reduction in odds of 30‐day mortality (mOR 0.86; 95%CI 0.51–1.44). Survival analysis with time‐varying TXA found a non‐significant 24% reduction in the hazard of death within 30‐day of injury (HR 0.76; 95%CI 0.41–1.38). In terms of morbidity, TXA‐treated patients had significantly lower odds of MOF compared to untreated patients (mOR 0.71; 95%CI 0.53–0.95) and a non‐significant reduction in odds of poor recovery (mOR 0.89; 95%CI 0.68–1.18).

**TABLE 3:** Adjusted marginal odds ratios (mOR) comparing primary and secondary outcomes among patients who received TXA within 3 h to those who did not.

Outcome	mOR (95% CI)
Mortality	
48‐h	0.74 (0.61, 0.90)
72‐h	0.67 (0.61, 0.74)
7‐day^a^	0.78 (0.62, 0.98)
30‐day	0.86 (0.51, 1.44)
Morbidity	
MOF	0.71 (0.53, 0.95)
Poor recovery	0.89 (0.68, 1.18)

Abbreviations: mOR; marginal odds ratio; MOF multi‐organ failure.

^a^ Primary outcome.

Receiving TXA within 2 h of injury was associated with a 27% reduction in odds of 7‐day mortality (mOR 0.73 95%CI 0.61–0.86) and non‐significant reduced odds of mortality were found for 48‐h mortality, 30‐day mortality, and MOF (Table 4). We could not create balanced IPTW weights for patients receiving TXA within 1 h due to the small sample size in the treatment group. After excluding all CTD deaths, the effect estimates for TXA administration were consistent in direction and magnitude with the main analysis (Table 4). Removal of severe TBI patients also resulted in unbalanced models due to the small number of mortality outcomes.

**TABLE 4:** Planned sub‐group analysis marginal odds ratios (and 95% confidence intervals).

Outcomes	TXA within 2 h of injury (No TXA *n* = 524, TXA (2h) *n* = 88)	Removal of CTD (No TXA *n* = 508, TXA (3h) *n* = 127)
	mOR (95% CI)	mOR (95% CI)
Mortality		
48‐h	0.84 (0.63, 1.10)	0.75 (0.57, 0.98)
72‐h	0.77 (0.59, 0.99)	0.67 (0.57, 0.78)
7‐day^a^	0.73 (0.61, 0.86)	0.79 (0.60, 1.04)
30‐day	0.90 (0.49, 1.67)	0.87 (0.50, 1.51)
Morbidity		
MOF	0.86 (0.60, 1.23)	0.71 (0.53, 0.95)
Poor recovery	0.94 (0.63, 1.41)	0.90 (0.68, 1.19)

Abbreviations: CI, confidence intervals; CTD, catastrophic tissue destruction; MOF multi‐organ failure; mOR, marginal odds ratio; TXA, tranexamic acid.

^a^ Primary outcome.

In a sensitivity analysis stratifying our population by injury force type, we observed non‐significant reduced odds of 7‐day mortality for all three force types: (blunt: mOR 0.78; 95%CI, 0.54–1.14; penetrating: mOR 0.82; 95%CI 0.62–1.08; blunt + penetrating: mOR 0.65; 95%CI 0.23–1.83). (Supporting Information S2).

Our final series of sensitivity analyses stratified patients by injury severity (Table 5). First we stratified the entire population of patients with either severe hemorrhage or severe TBI or if the maximal severity of hemorrhage or TBI was moderate. There was no significant difference in 7‐day mortality among either the severe (mOR 0.73; 95%CI 0.45–1.18) or mild/moderate (mOR 1.03; 95%CI 0.49–2.15) groups. There was significantly reduced odds of 48‐h and 72‐h mortality and MOF in patients with severe hemorrhage + TBI.

**TABLE 5:** Sensitivity analysis marginal odds ratios for severe and mild or moderate hemorrhage + TBI.

Outcomes	Severe hemorrhage + TBI (*n* = 302)	Mild/Moderate hemorrhage + TBI (*n* = 354)
	mOR (95% CI)	mOR (95% CI)
Mortality		
48‐h	0.73 (0.56, 0.95)	0.76 (0.15, 3.90)
72‐h	0.70 (0.57, 0.86)	0.43 (0.06, 2.99)
7‐day^a^	0.73 (0.45, 1.18)	1.03 (0.49, 2.15)
30‐day	0.72 (0.35, 1.47)	1.24 (0.54, 2.81)
Morbidity		
MOF	0.60 (0.44, 0.82)	0.71 (0.30, 1.66)
Poor recovery	0.70 (0.34, 1.45)	1.17 (0.97, 1.40)

Abbreviations: CI, confidence intervals; MOF multi‐organ failure; mOR, marginal odds ratio; TBI, traumatic brain injury.

^a^ Primary outcome.

We then stratified the entire population by severity of hemorrhage, defined as mild/moderate hemorrhage (SI < 1.2) or severe hemorrhage (SI ≥ 1.2) (Table 6). We observed a significant reduction in 48‐h mortality in both groups and a significant reduction in 72‐h mortality and MOF in the mild/moderate hemorrhage groups. Lastly, we stratified the entire population of patients by severity of TBI. (Supporting Information S1) In severe TBI we observed significant reductions in mortality at 48‐h, 72‐h, and 7‐day with the largest benefit at 72‐h (mOR 0.41; 95%CI 0.37–0.46). Within the mild/moderate TBI group there were very large confidence intervals and a trend toward increased mortality at 48‐h, 72‐h and 7‐day mortality that changed to a non‐significant reduction in mortality at 30‐day (mOR 0.89; 95%CI 0.43–1.82).

**TABLE 6:** Sensitivity analysis marginal odds ratios for severe and mild or moderate hemorrhage.

Outcomes	Hemorrhage		TBI	
	Severe (SI ≥ 1.2) *n* = 128	Mild/Moderate (SI < 1.2) *n* = 599	Severe *n* = 291	Mild/Moderate *n* = 365
	mOR (95% CI)	mOR (95% CI)	mOR (95% CI)	mOR (95% CI)
Mortality				
48‐h	0.62 (0.53, 0.73)	0.79 (0.65, 0.97)	0.47 (0.42, 0.53)	3.35 (1.41, 7.93)
72‐h	0.78 (0.58, 1.05)	0.66 (0.61, 0.71)	0.41 (0.37, 0.46)	2.34 (1.41, 3.89)
7‐day^a^	0.65 (0.38, 1.13)	0.83 (0.67, 1.01)	0.53 (0.37, 0.75)	1.53 (1.05, 2.23)
30‐day	0.80 (0.38, 1.66)	0.89 (0.55, 1.44)	0.72 (0.31, 1.68)	0.89 (0.43, 1.82)
Morbidity				
MOF	0.82 (0.58, 1.16)	0.69 (0.50, 0.97)	0.45 (0.15, 1.30)	0.56 (0.37, 0.86)
Poor recovery	0.65 (0.21, 1.97)	0.95 (0.83, 1.09)	0.87 (0.34, 2.21)	0.79 (0.66, 0.95)

Abbreviations: CI, confidence intervals; MOF multi‐organ failure; mOR, marginal odds ratio; SI, shock index; TBI, traumatic brain injury.

^a^ Primary outcome.

## 4 Discussion

A prior study among a hemorrhage‐only cohort of patients from the EpiC study found significant associations between mortality and TXA administration with 38% reduced mortality at 24‐h and 37% reduction at 30‐day [33] In this study, we examine whether the benefit of TXA extends to patients with concurrent hemorrhage + TBI by assessing the association between TXA administered within 3 h on mortality and morbidity. We found a statistically and clinically significant 22% reduction in the odds of mortality at 7 days. There was also a statistically significant reduction in secondary outcomes of 48‐ and 72‐h mortality, but not in 30‐day mortality. In the TXA‐treated group, there was a significantly reduced odds of MOF and a non‐significant trend toward reduced odds of poor recovery. Subgroup and sensitivity analyses by timing of TXA administration and hemorrhage severity were consistent with the primary findings of reduced mortality and mortality. The sensitivity analysis by TBI severity found reduced odds of mortality and morbidity in severe TBI but increased odds of early mortality in mild and moderate TBI.

This study's findings are consistent with the current literature. One example is the PATCH‐Trauma trial conducted in Australia, New Zealand, and Germany which found significantly reduced mortality in patients with both hemorrhage and TBI at 24‐h (HR 0.69) and 28‐day (HR 0.79) [27] Karl et al. also completed a meta‐analysis that found reduced 1‐month mortality with TXA administration in patients with hemorrhage + TBI (risk ratio, 0.83; 95%CI, 0.71–0.97) [30]. Our sensitivity analysis that found benefit in severe (mOR 0.53) but not mild/moderate TBI (mOR 1.53) are opposite of most studies on TXA for TBI, most recently summarized in a meta‐analysis by Monge et al. [26].

Regarding the timing of TXA administration, multiple randomized trials and meta‐analyses demonstrate that TXA confers its strongest mortality benefit when given early, ideally within 1 hour after injury [22, 23]. This study found a 27% reduction in mortality at 7‐day when given within 2 h compared to 22% when given within 3 hours. Previous studies have reported mixed 30‐day mortality outcomes, the PATCH‐Trauma trial found significant 28‐day mortality reduction whereas several meta‐analyses found that the early survival advantage from TXA does not consistently extend to 28–30‐day mortality [27, 30, 46, 47]. Factors that predispose to late death after successful early resuscitation include trauma‐induced coagulopathy, prolonged shock and ischemia, massive transfusion and transfusion‐related complications, and nosocomial infection progressing to sepsis and multi‐organ failure [10, 48]. At later endpoints such as 30‐day, deaths secondary to these pathophysiologic mechanisms may epidemiologically overshadow early survival benefits gained from effective early resuscitation. We were unable to study TXA delivered at 1 h due to a limited sample size.

Long‐term neurological outcomes are an important indicator of treatment effectiveness in hemorrhage + TBI patients due to the high rates of morbidity among survivors. Across the entire cohort and across all subgroups, TXA was non‐significantly associated with higher odds of good recovery at discharge (GOSE ≥ 7). A likely contributing factor to our non‐significant findings is the high, median discharge GCS of 15 in both treatment groups, limiting the ability to detect an improvement or change given the low incidence of poor neurologic outcomes. Our findings are consistent with other studies that found no improvement in GOSE as a primary outcome after TXA administration [29, 49, 50, 51]. Studies of TXA in TBI have measured intracerebral hemorrhage volumes but found minimal to no impact despite the known antifibrinolytic mechanisms of TXA [23, 28, 52]. Another potential mechanism for why TXA may improve neuro outcomes are the anti‐inflammatory effects which have been measured in other bleeding populations but are still not well understood in TBI [14, 53].

### 4.1 Limitations

Although our study reflects one of the largest studies of TXA for concurrent hemorrhage + TBI to date, our sample size was relatively small, especially for some subgroup and sensitivity analyses, leading to large confidence intervals and limited power to detect statistically significant effects. Additionally, this observational dataset relies on chart review and abstraction of clinical variables, which can lead to missing data. However, the EpiC study is not heavily affected by missing data and data are collected by clinically trained data abstractors that follow a rigorous and standardized methodology. Finally, our findings suggest a pattern of confounding by indication, where patients with the most severe injuries are both most likely to receive TXA and most likely to suffer adverse outcomes. Although we mitigated this bias using IPTW and doubly robust estimation, there is potential that additional unobserved covariates result in residual unmeasured confounding.

## 5 Conclusion

Our findings demonstrate that TXA delivered within 3 h post‐injury was associated with a significant 22% reduction in 7‐day mortality and reduced odds of MOF or death among those with hemorrhage + TBI. Results from this study support early administration of TXA to patients with hemorrhage + TBI. Further research is needed to assess the effectiveness within specific subgroups of hemorrhage + TBI patients.

## Author Contributions

**Adane F. Wogu:** writing – original draft (co‐lead), writing – review and editing (co‐lead), formal analysis (lead), validation (lead), visualization (equal), methodology (equal). **Julia Dixon:** writing – original draft (co‐lead), writing – review and editing (co‐lead), methodology (equal), supervision (co‐lead), project administration (co‐lead), funding acquisition (co‐lead), and investigation (lead). **Maria D. Rodriguez:** data curation (lead), writing – original draft (supporting), writing – review and editing (supporting). **Dale Barnhart:** writing – review and editing (supporting). **Rachel Patel:** writing – original draft (supporting), writing – review and editing (supporting). **Hendrick Lategan:** supervision (supporting), project administration (co‐lead), funding acquisition (supporting), writing – review and editing (supporting). **George Oosthuizen:** writing – review and editing (supporting). **Janette Verster:** supervision (co‐lead), writing – review and editing (supporting). **Shaheem de Vries:** supervision (co‐lead), project administration (co‐lead), writing – review and editing (supporting), funding acquisition (supporting). **Craig Wylie:** supervision (co‐lead), project administration (co‐lead), writing – review and editing (supporting). **EpiC Study Site Collaborators:** project administration (supporting), supervision (co‐lead), resources (supporting), writing – review and editing (supporting). **Elaine Erasmus:** writing – review and editing (supporting). **Steven G. Schauer:** funding acquisition (supporting), conceptualization (co‐lead), writing – review and editing (supporting). **Nee‐Kofi Mould‐Millman:** supervision (co‐lead), conceptualization (lead), writing – review and editing (co‐lead), resources (lead), methodology (supporting), project administration (lead), methodology (equal), funding acquisition (lead). The group author “EpiC Study Site Collaborators” is comprised of: Mohammed Mayet, MBChB‐Khayelitsha Hospital; Lesley Hodsdon, MBChB‐Worcester Regional Hospital' L'Oreal Snyders, MBChB‐Delft Community Centre; Leigh Wagner, MBChB‐Khayelitsha Site B Community Health Centre; Karlien Doubell, MBChB‐Ceres District Hospital; Denise Lourens, MBChB‐Worcester Forensic and Pathology Services Laboratory. All are employees of Western Cape Government Health and Wellness, Cape Town, South Africa.

## Funding

United States Defense Health Agency award numbers/grant log numbers: W81XWH‐22‐1–0883/RC210170, W81XWH‐20‐2–0042/BA190049, HT9425‐23‐1–1025/PR220453, ID07200010‐301.

## Consent

A waiver of informed consent was used for this study as approved by the primary IRB of record, Stellenbosch University Human Research Ethics Committee (project ID 14866, Reference Number N20/03/036). Colorado Multiple Institute Review Board relied on the Stellenbosch IRB (COMIRB #20–2176) and a concurrence memo from the Defense Health Agency Office of Human Research Oversight with the U.S. Department of Defense's subject protection requirements (OHRO Log #E01863.1x).

## Conflicts of Interest

The authors declare no conflicts of interest.

## Supporting information


Supporting Information S1

Supporting Information S2

## Data Availability

Public access to the dataset is closed. The datasets included and/or analyzed during the current study are available from the corresponding author upon reasonable request.
