# Supplementary material for: Mortality Benefit of Tranexamic Acid for Hemorrhage With Concurrent Traumatic Brain Injury: Outcomes From a Prospective Cohort Study in a High‐Trauma, Prolonged Care Setting
Source: World J Surg. 2025 Nov 28;50(1):233–43. doi: 10.1002/wjs.70161 (PMC12831529; doi:10.1002/wjs.70161)
Supplement: Supplementary file 1 — Supporting Information S1 [file WJS-50-233-s002.docx]

Mortality Benefit of Tranexamic Acid for Hemorrhage with Concurrent Traumatic Brain Injury: Outcomes from a Prospective Cohort Study in a High-Trauma, Prolonged Care Setting

World Journal of Surgery

Adane F Wogu PhD; Julia M Dixon, MD, MPH; Maria D Rodriguez, MPH; Dale Barnhart, ScD; Rachel Patel, MD; Hendrick J. Lategan, MBChB; Willem Stassen, PhD; Elaine Erasmus, MBChB; Shaheem de Vries, MBChB; George Oosthuizen, MBChB, PhD; Craig Wylie, MPhil; Janette Verster, MBChB; EpiC Study Site Collaborators; Steve Schauer, DO, MS; Nee-Kofi Mould-Millman, MD, PhD, MSCS;

Corresponding Author: Julia M Dixon, MD, MPH.

Affiliation: Department of Emergency Medicine, School of Medicine, University of Colorado Denver.

Email: Julia.Dixon@cuanschutz.edu

**Inclusion and Exclusion Criteria**

**Any Hemorrhage Criteria**

- Blunt or penetrating injury or mixed blunt + penetrating injury force type AND AIS Body >= 1 (exclude head and face) AND at least one of the following
- SBP <=100 (first hospital and/or primary EMS) OR Shock index >= 1.0 (first hospital and/or primary EMS) OR SOFA cardiovascular score of >=3 on day 1 OR Initial lactate >= 4.0 OR Initial base deficit >=6 OR Initial pH <= 7.2 OR Administration of TXA in the initial 24 hours post injury OR Receipt of vasopressors, inotropes, or blood products within first 24 hours from injury OR Circulatory diagnoses in first 24 hours (Shock, Hemorrhagic hypovolemia, Cardiac Tamponade, Cardiac arrest) OR Died from hemorrhage

**Stratification**

Stratification begins with the **severe** category. A patient is classified as **severe** if they meet **at least one severe criterion**. If no severe criteria are met, the patient is evaluated for the **moderate** category. Meeting **at least one moderate criterion** results in classification as **moderate**. Patients who do not meet any severe or moderate criteria are classified as **mild**.

**Online Resource Table 1** Hemorrhage severity stratification

| **Criteria** | **Mild** | **Moderate** | **Severe** |
| --- | --- | --- | --- |
| *Systolic Blood Pressure* | >90 mmHg | >70 – ≤ 90 mmHg | ≤ 70 mmHg |
| *Shock Index* | ≥ 0.7 – <1.2 | ≥1.2 – < 1.6 | ≥1.6 |
| *Base Deficit* | <6mmol/l | ≥6 - <10mmol/l | ≥ 10mmol/l |
| *Lactate* | --- | --- | ≥ 10 mmol/l |
| *Hemoglobin* | > 7 g/l | >5 – ≤7 g/l | ≤ 5 g/l |
| *Packed Red Blood Cells* | < 3 units | ≥3 – <5 units | ≥ 5 units |
| *Whole Blood* | 0 units | >0 – <3 units | ≥ 3 units |
| *Fresh Frozen Plasma* | 0 units | >0 – <3 units | ≥ 3 units |
| *Freeze Dried Plasma* | <2 units | ≥2 – <4 units | ≥ 4 units |
| *Platelets* | 0 units | ≤ 1 unit | > 1 unit |
| *Cryoprecipitate* | 0 units | ≤ 1 unit | > 1 unit |
| *Vasopressors* | --- | --- | ≥1 unit/24 hrs |
| *Inotropes* | --- | --- | ≥1 unit/24 hrs |

### **Any TBI Criteria:**

- Blunt or penetrating injury or mixed blunt + penetrating injury force type AND AIS head >=2 OR Death from TBI

**Online Resource Table 2** TBI Severity Stratification

| **Criteria** | **Mild ‘Any TBI’** | **Moderate ‘Any TBI’** | **Severe ‘Any TBI’** |
| --- | --- | --- | --- |
| GCS Score | 13 – 15 | 9 – 12 | ≤8 |
| Abnormal CT Brain Severe: diffuse axonal injury or shift >5mm or herniation |  |  | + |
| Abnormal CT Brain Moderate: Intracranial bleeding or skull fracture or parenchymal injury |  | + | +/- |

**Online Resource Table 3** Imputation of Missing GODS

| **Discharge GCS** | **Imputed GODS** |
| --- | --- |
| 3 | 2, vegetative state |
| 4-6 | 3, lower severe disability |
| 7-9 | 4, upper severe disability |
| 10-11 | 5, lower moderate disability |
| 12-13 | 6, upper moderate disability |
| 14 | 7, lower good recovery |
| 15 | 8, upper good recovery |

**Online Resource Table 4** Variables used in the IPTW model versus the weighted multivariable logistic regression model

| **Covariate** | **Format** | **IPTW analysis** **(PS model)** | **Outcome analysis** **(WMVLR^1^ model)** |
| --- | --- | --- | --- |
| Sex | categorical (2) | Yes | Yes |
| Age | continuous | Yes | Yes |
| Injury force type | categorical (3) | No | No |
| Injury mechanism | categorical (5) | Yes | Yes |
| Shock index | continuous | Yes | Yes |
| SBP | categorical (3) | No | No |
| NISS | categorical (3) | Yes | Yes |
| GCS^2^ | categorical (4) | Yes | Yes |
| Blood products before treatment | categorical (2) | Yes | Yes |
| Blood products after treatment | categorical (2) | No | Yes |
| Time from injury to first facility | categorical (2) | Yes | Yes |
| AIS: head | categorical (3) | Yes | No |
| AIS: neck & torso | categorical (4) | Yes | No |
| AIS: all other reasons | categorical (4) | Yes | No |
| Time from injury to treatment | continuous | No | No |
| 1 *Weighted multivariable logistic regression*  2 *One of the four categories is “Missing” as there are 10 patients with missing GCS* | | | |
