# Supplementary material for: Mortality Benefit of Tranexamic Acid for Hemorrhage With Concurrent Traumatic Brain Injury: Outcomes From a Prospective Cohort Study in a High‐Trauma, Prolonged Care Setting
Source: World J Surg. 2025 Nov 28;50(1):233–43. doi: 10.1002/wjs.70161 (PMC12831529; doi:10.1002/wjs.70161)
Supplement: Supplementary file 2 — Supporting Information S2 [file WJS-50-233-s001.docx]

Mortality Benefit of Tranexamic Acid for Hemorrhage with Concurrent Traumatic Brain Injury: Outcomes from a Prospective Cohort Study in a High-Trauma, Prolonged Care Setting

World Journal of Surgery

Adane F Wogu PhD; Julia M Dixon, MD, MPH; Maria D Rodriguez, MPH; Dale Barnhart, ScD; Rachel Patel, MD; Hendrick J. Lategan, MBChB; Willem Stassen, PhD; Elaine Erasmus, MBChB; Shaheem de Vries, MBChB; George Oosthuizen, MBChB, PhD; Craig Wylie, MPhil; Janette Verster, MBChB; EpiC Study Site Collaborators; Steve Schauer, DO, MS; Nee-Kofi Mould-Millman, MD, PhD, MSCS;

Corresponding Author: Julia M Dixon, MD, MPH.

Affiliation: Department of Emergency Medicine, School of Medicine, University of Colorado Denver.

Email: [Julia.Dixon@cuanschutz.edu](mailto:Julia.Dixon@cuanschutz.edu)

**Online Resource Table 5** Planned sensitivity analysis by injury force type

| **Outcomes** | **Blunt**  No TXA n=367, TXA(3h) n=84 | | **Penetrating**  No TXA n=88, TXA(3h) n=33 | **Blunt and Penetrating** No TXA n=69, TXA(3h) n=15 |
| --- | --- | --- | --- | --- |
| **Mortality** | **mOR (95% CI)** | **mOR (95% CI)** | | **mOR (95% CI)** |
| 48-hour | 0.77 (0.56, 1.05) | 0.73 (0.52, 1.04) | | 0.54 (0.32, 0.93) |
| 72-hour | 0.67 (0.55, 0.81) | 0.73 (0.54, 0.98) | | 0.50 (0.24, 1.03) |
| 7-day^a^ | 0.78 (0.54, 1.14) | 0.82 (0.62, 1.08) | | 0.65 (0.23, 1.83) |
| 30-day | 0.79 (0.48, 1.28) | 0.67 (0.35, 1.28) | | 2.00 (0.99, 4.03) |
| **Morbidity** |  |  | |  |
| MOF | 0.66 (0.53, 0.81) | 0.68 (0.41, 1.12) | | 1.06 (0.52, 2.14) |
| Poor recovery | 0.88 (0.72, 1.06) | 0.58 (0.32, 1.08) | | 1.79 (0.84, 3.82) |

^a^Primary outcome. TXA, tranexamic acid; CTD, catastrophic tissue destruction; mOR, marginal odds ratio; CI, confidence interval; SOFA, sequential organ failure assessment
